# The Autoimmune Tautology: From Polyautoimmunity and Familial Autoimmunity to the Autoimmune Genes

**DOI:** 10.1155/2012/297193

**Published:** 2012-05-20

**Authors:** Juan-Manuel Anaya, Adriana Rojas-Villarraga, Mario García-Carrasco

**Affiliations:** ^1^Center for Autoimmune Diseases Research (CREA), School of Medicine and Health Sciences, Universidad del Rosario, Carrera 24 No. 63-C-69, Bogota, Colombia; ^2^Systemic Autoimmune Diseases Research Unit, Hospital General Regional No. 36, IMSS and Rheumatology Department, School of Medicine, Benemérita Universidad Autónoma de Puebla, Puebla P4E, Mexico

Autoimmune diseases (ADs) are chronic conditions initiated by the loss of immunological tolerance to self-antigens and represent a heterogeneous group of disorders that afflict specific target organs or multiple organ systems [1]. The chronic nature of these diseases places a significant burden on the utilization of medical care, direct and indirect economic costs, and quality of life. The fact that ADs share several clinical signs and symptoms (i.e., subphenotypes), physiopathological mechanisms, and genetic factors has been called autoimmune tautology and indicates that they have common mechanisms [2–8].

In clinical practice, there are two conditions supporting this theory: polyautoimmunity and familial autoimmunity. Polyautoimmunity is the presence of two or more ADs in a single patient while familial autoimmunity occurs when different relatives from a nuclear family present with diverse ADs [4]. These conditions indicate that similar genetic, epigenetic, and environmental factors influence ADs [8].

In rhetoric, tautology (from Greek tauto, “the same” and logos, “word/idea”) is an obvious statement. In medical practice, Sjögren's syndrome could be considered the “autoimmune diabetes” or the “celiac disease” of the salivary and lachrymal glands. In logic, tautology is a formula, which is true in every possible interpretation. Thus, autoimmune tautology means that an AD is similar to a second one, to a third one, and so on. Its formula is Vpq = AD_1_≃AD_2_≃AD_3_, where Vpq represents the symbol of tautology. ADs cannot be all identical because the target cell and the affected organ may differ from one AD to another one. In addition, trigger factors as well as the age at onset may vary among them and from one patient to another. Yet, autoimmune mechanisms of injury may be common including predisposing and protective factors. One step forward to the demonstration of this logically valid formula will be a new taxonomy of ADs based on common and specific subphenotypes that will allow us to predict and prevent them, tailor individual medical decisions, and provide personalized healthcare while facilitating patient's participation in their treatment and eventual cure of their disease.

In Table 1, ten shared characteristics supporting the autoimmune tautology are summarized. In this special issue of *Autoimmune Diseases, *a dozen of papers are devoted to these characteristics. Evolution and genetics of ADs, including the biological significance of evolution in autoimmune phenomena, a meta-analysis of HLA class II in six ADs in Latin America, genetic factors of autoimmune thyroid diseases in Japanese, and an *in silico* approach of the autoimmune tautology are included. An updated review on epigenetics and ADs is also presented.

Environmental factors play an important role in the induction of ADs. This special issue also contains a very interesting hypothesis about local cartilage trauma as a pathogenic trigger factor of autoimmunity, a review about the induction of autoimmunity by microbial infections, and another one on the effect of selenium on HLA-DR expression of thyrocytes. How does age at onset influence the outcome of ADs is also reviewed, and an update on lupus nephritis is offered.

Last but not least, a careful analysis of concomitant ADs in patients with systemic lupus erythematosus, rheumatoid arthritis (RA), multiple sclerosis (MS), and systemic sclerosis is reported. Polyautoimmunity is the term proposed for this association of disorders, which encompasses the concept of a common origin for these diseases. A lack of association between spondyloarthropathies (SpAs) and ADs is described, highlighting the fact that SpAs correspond more to autoinflammatory diseases rather than to ADs.

We hope that readers of *Autoimmune Diseases* will find in this special issue not only accurate data and updated reviews on the common mechanisms of ADs, but also important questions to be resolved such as their missing heritability, the antagonisms of some disorders (i.e., RA and MS), their prevention, the effect of ethnicity, socioeconomic status and health care system on their outcome, and the role of the autoimmunologist, among others.

*Juan-Manuel Anaya* *Adriana Rojas-Villarraga* *Mario García-Carrasco*

## Figures and Tables

**Table 1:** Shared characteristics among autoimmune diseases (ADs) supporting the autoimmune tautology*.

Characteristic	Comment
Female predominance	The more frequent the AD and the later it appears, the more women are affected.

Similar pathophysiology	Damage induced by T or B cells, or both, plays a major pathogenic role in ADs. Although the autoimmune phenotype varies depending on the target cell and the affected organ, the local mechanisms for tissue injury are similar.

Shared subphenotypes	Mathematical approaches for precisely defining subphenotypes based on accurate clinical and immunological databases, combined with strengthening molecular genetics analyses, have significant promise for a better understanding of ADs.

Age at onset influences severity	Early age at onset is a poor prognostic factor for some ADs.

Similar environmental factors	Although a latitudinal gradient of infectious agents exists, Epstein-Barr virus and cytomegalovirus are notorious as they are consistently associated with multiple ADs. Some infections could be protective against ADs development. Smoking has also been consistently associated with several ADs.

Ancestry influences clinical presentation	Amerindian ancestry influences the risk of acquiring ADs as well as its severity.

Common genetic factors	The genetic risk factors for ADs consist of two forms: those common to many ADs and those specific to a given disorder. Combinations of common and disease-specific alleles at HLA and non-HLA genes in interaction with epigenetic and environmental factors over time will determine the final phenotype.

Polyautoimmunity	Factors significantly associated with polyautoimmunity are female gender and familial autoimmunity.

Familial autoimmunity	Unlike familial AD, which corresponds to the presence of one specific AD in various members of a nuclear family, familial autoimmunity uses the term “autoimmune disease” as a trait that encompasses all accepted pathologies for which evidence suggests an autoimmune origin.

Similar treatment	Similar biological and nonbiological therapies are used to treat diverse ADs.

*Adapted from references [2–8].
